# An early Eocene fish assemblage associated with a barite deposit in the lower part of the Crescent Formation, Olympic Peninsula, Washington State, USA

**DOI:** 10.1007/s12542-024-00692-y

**Published:** 2024-07-09

**Authors:** James L. Goedert, Steffen Kiel, Eric J. Thomas, Jürgen Kriwet

**Affiliations:** 1 Burke Museum of Natural History and Culture, University of Washington, Seattle, WA 98195 USA; 2 Department of Palaeobiology, Swedish Museum of Natural History, Box 50007, 10405 Stockholm, Sweden; 3 4013 47th Avenue SW, Seattle, WA 98116 USA; 4 Faculty of Earth Sciences, Geography and Astronomy, Department of Palaeontology, UZA 2, Geocentre, University of Vienna, Josef-Holaubek-Platz 2, 1090 Vienna, Austria

**Keywords:** Ypresian, *Chlamydoselachus*, *Notorynchus*, *Isistius*, *Mitsukurina*, *Otodus*, *Alopias*, *Egertonia*

## Abstract

Abundant shark and rare actinopterygian teeth are reported from a locality within the early Eocene (Ypresian) lower part of the Crescent Formation exposed in the Hamma Hamma River valley on the eastern Olympic Peninsula, Washington State, USA. This part of the Crescent Formation is predominantly submarine volcanic basalt with some sedimentary interbeds deposited in deep water. The teeth are derived from sediments that appear to directly overlay and in places interfinger with the margins of an anomalous lenticular barite deposit; one tooth was found in the barite. Genera represented include deep-water taxa (aff. *Chlamydoselachus*, *Mitsukurina*, *Notorynchus*, *Odontaspis*) and open marine, epipelagic sharks (*Alopias*, *Brachycarcharias*, *Jaekelotodus*, *Macrorhizodus*, *Otodus*, *Striatolamia*). The only other fossils found were two fragmentary shark vertebrae, numerous shark dermal ossicles, a single teleost tooth (*Egertonia*) and abundant, minute valves of a discinid brachiopod. This is the first report of macrofossils from the lower part of the Crescent Formation and the only early Eocene shark assemblage described from the North Pacific Basin. The shark assemblage also corroborates paleodepositional interpretations of the lower Crescent Formation as being in part ancient volcanic seamounts during early Eocene time.

## Introduction

Early Eocene fishes are well known from Europe, Africa, and eastern North America (e.g., Kent 1999; Adnet and Cappetta 2008; Adnet et al. 2008; Iserbyt and De Schutter 2012; Padilla et al. 2014; Marramà et al. 2018, 2019; Popov and Lopyrev 2023, and references therein), however, there are surprisingly few reports of fishes from early Eocene strata of the Pacific Basin. They are limited to only a few incidental records and preliminary reports from the Chatham Islands and Australia in the south (Keyes 1987; Kemp 1991) and western North America in the north (e.g., Squires and Goedert 1994; Gonzalez-Barba 2004). Therefore, any additional data on early Eocene fishes from the North Pacific Basin is of significance in understanding distributions of various taxa.

On the Olympic Peninsula in western Washington State, early and middle Eocene molluscan fossils have been recorded from several localities in the uppermost part of the Crescent Formation (e.g., Arnold 1906; Weaver and Palmer 1922; Squires et al. 1992; Squires and Goedert 1994). However, except for foraminiferans (Pardee 1921; Rau 1964), fossils have not been previously reported from the lower part of the Crescent Formation. There are few previous reports of fossil shark teeth from the Crescent Formation. The earliest that we are aware of is Park (1946: p. 308) who stated shark teeth had been found at “three widely separated localities” associated with volcanic rocks, although he did not disclose what part of the Crescent Formation these localities were in, nor did he mention a repository for the specimens. Two shark teeth were recorded by Squires and Goedert (1994) from the upper part of the Crescent Formation on the south side of the Olympic Mountains. Kočí et al. (2022) mentioned shark teeth from early Eocene rocks of the Crescent Formation in the Black Hills near Olympia, and an unidentified shark tooth was reported from the middle Eocene ‘Crescent/McIntosh transition zone’ in the Doty Hills (Squires and Goedert 1995) of southwestern Washington.

The purpose of this paper is to document a previously unknown low diversity fish assemblage based on teeth and dermal ossicles found in the early Eocene lower unit of the Crescent Formation, in marine deep-water sediments associated with a small, anomalous barite deposit.

### Geological setting

In western Washington State, the basement strata are the Crescent Formation (Arnold 1906), part of Siletzia, a terrane comprised of submarine volcanic rocks accreted to North America in Eocene time (e.g., Wells et al. 2014; Eddy et al. 2017; Anderson et al. 2024). The Crescent Formation is predominantly submarine tholeiitic basalt, and just to the north of the study area its thickness is as much as 15 km (Cady et al. 1972a), making it one of the thickest exposed sections of submarine volcanic rocks anywhere in the world. The formation has been informally divided into an upper and lower unit (Cady et al. 1972a; Cady 1975; Tabor and Cady 1978); the lower unit is pillow and massive basalt and associated deep water sediments, and the upper unit is pillow basalt with subaerial basalt flows in its upper part. Sediments of the upper unit were deposited in a warm, upper bathyal to upper neritic marine environment based on foraminiferans and mollusks (e.g., Rau 1966; Squires et al. 1992) and preserve wood fragments (Kiel 2008).

**Fig. 1:**
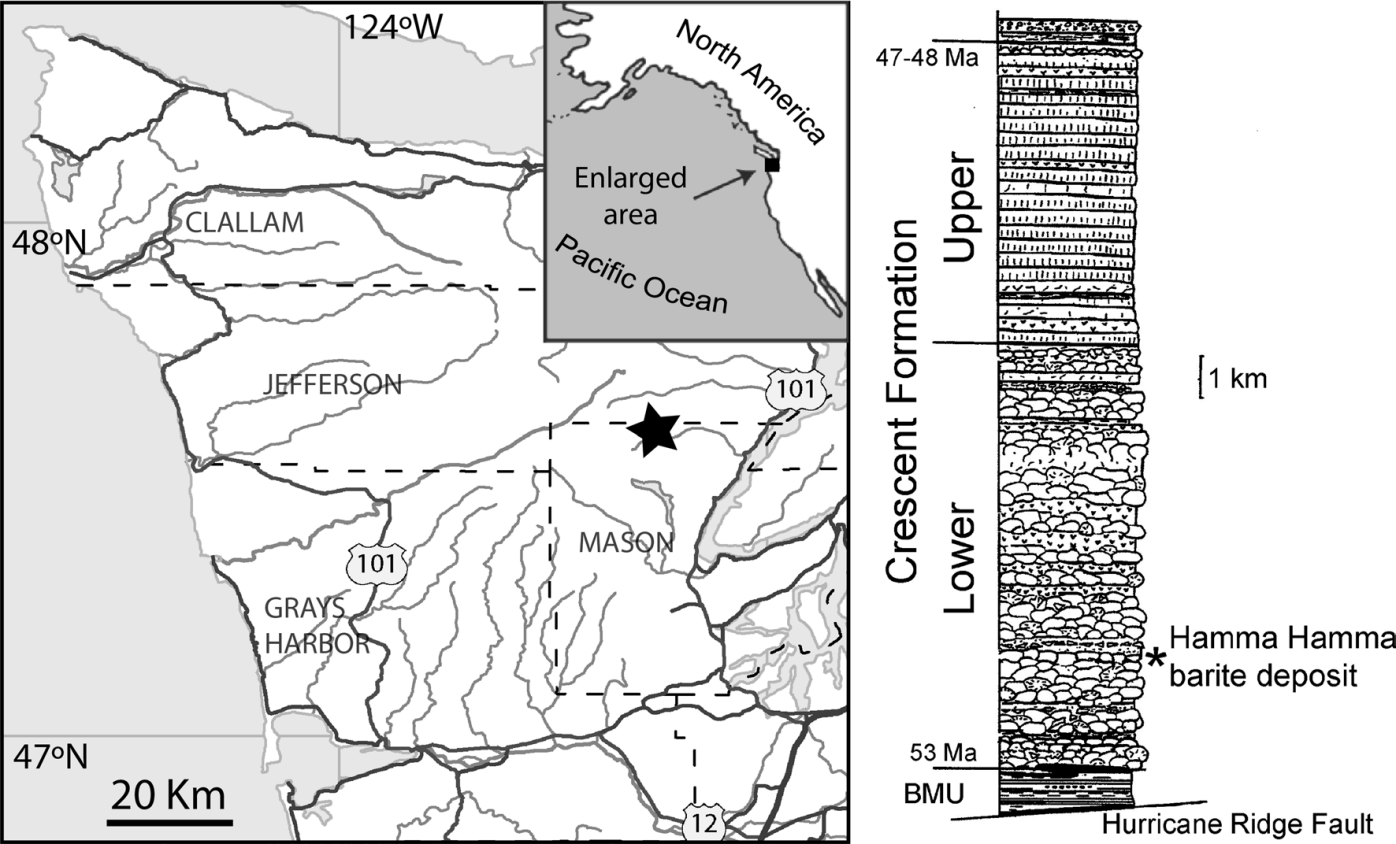
Map of the study area, Olympic Peninsula, Washington State, USA, and generalized stratigraphic position of the Hamma Hamma barite within the nearly 15 km thick Crescent Formation. BMU (Blue Mountain Unit) is comprised of younger rocks thrust below the Crescent Formation (Eddy et al. 2017). Geology modified from Babcock et al. (1992), age data compiled from Squires et al. (1992), Babcock et al. (1994), Wells et al. (2014) and Eddy et al. (2017)

The study area (Fig. 1) is in the Hamma Hamma River valley in the eastern Olympic Peninsula. An anomalous barite deposit (Hughes 1939) is deep within the lower unit of the Crescent Formation and has only been briefly mentioned in subsequent reports, listed as the ‘Maple Creek prospect’ (Park 1942; Green 1945; Moen 1964). It is shown as a manganese prospect on geologic maps (Cady et al. 1972a; Tabor and Cady 1978), even though manganese is apparently present only in trace amounts (Hughes 1939). Although Hughes (1939) refers to the barite deposit as a ‘vein’, it appears to be a lens-like body approximately 4.5 m thick and 46 m long (truncated by faulting and/or erosion at the western end, concealed at the eastern end) within sedimentary and volcanic deposits. The genesis of the barite deposit, speculated by Nisbet and Ambrose (1986) to be an ancient ‘white-smoker’ hydrothermal vent deposit (e.g., sensu Torres et al. 2003) is under investigation (J. Peckmann, pers. comm. 2021). Dips in the study area are steep, in places more than 80 degrees, generally toward the east or southeast (Cady et al. 1972a, b; Tabor and Cady 1978). At the northeastern end of the outcrop, the upper surface of the barite deposit is exposed on a cliff face as smooth spheroidal masses up to 1 m across. This uneven surface is capped by reddish colored mudstone with fish teeth, isolated matrix-supported and well-rounded pebbles, and pebbly sandstone approximately 15 cm thick, with another more indurated 13 cm of pebbly sandstone above that, continuous with sandstone, pebbly sandstone, and argillite with a combined thickness estimated to be 150 m (Hughes 1939). In places these sediments appear to interfinger with parts of the barite deposit. Most of the pebbles in the sandstone are 2–3 mm in their largest dimension although a few rare pebbles were found up to 15 mm across, and one was approximately 25 by 35 mm in diameter. The pebbles are angular to well-rounded. There are also small patchy layers of red mudstone, crystals and pebbles of barite, chlorite, and pyrite. Hematite is abundant in parts of the barite deposit as well as the associated sediments. The sediments directly above the barite preserve several fining-upward cycles and appear to be turbidites, with the coarsest material toward the barite, indicating the base is in the direction of the barite deposit. Exposed on the southwestern end of the outcrop and underlying the barite deposit are an undetermined thickness of basalt capped by manganese-stained, pebbly sediments less than 1 m thick, with some jasper-like material near the contact with barite. No fish teeth were observed in the sediments underlying the barite.

**Fig. 2:**
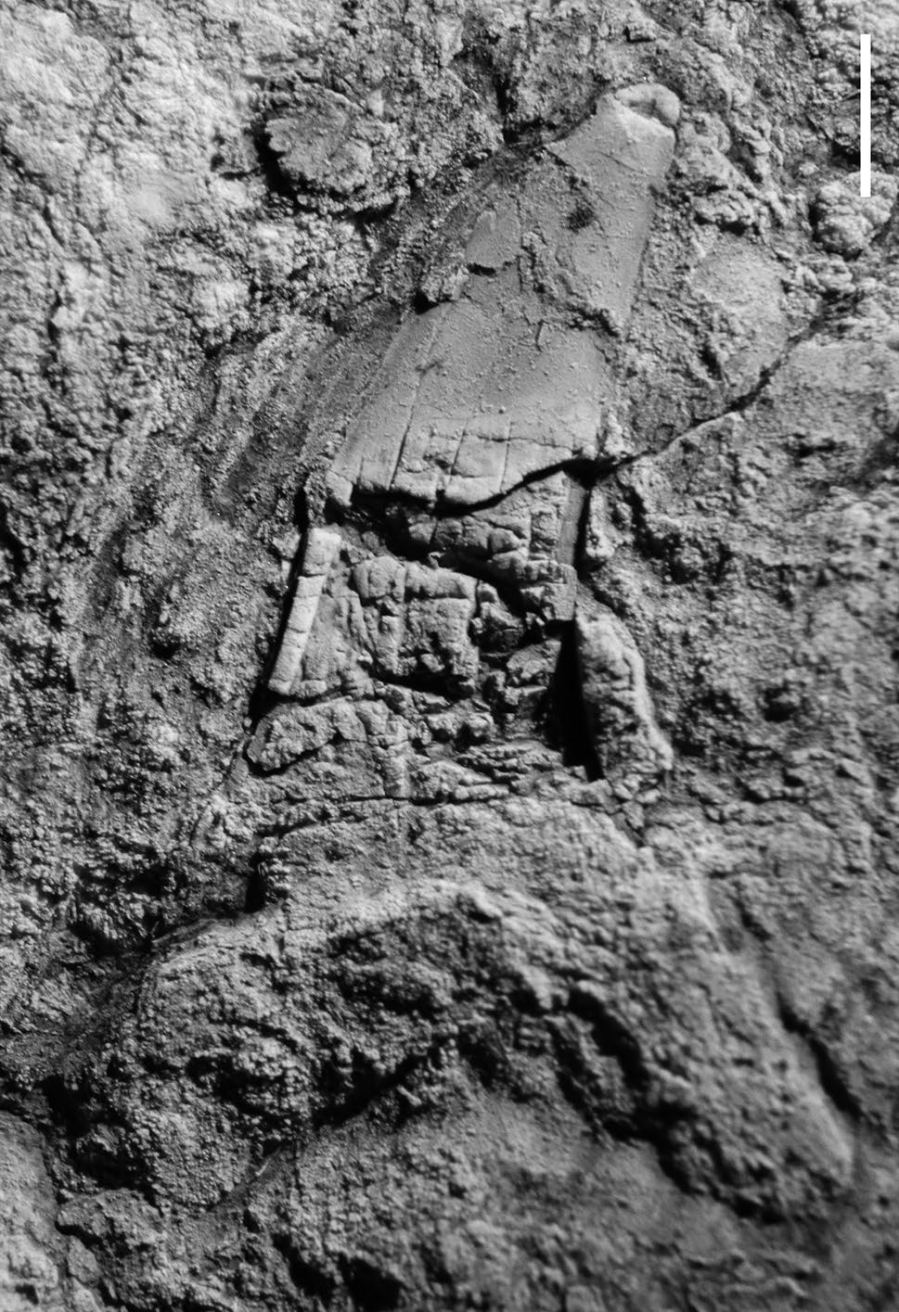
Weathered shark tooth (UWBMVP 124420) found in barite from the Hamma Hamma deposit. Scale bar is 3 mm

In the process of obtaining samples from the barite deposit, we unexpectedly found fossil fish teeth in sediments directly associated with and stratigraphically above the barite. Contra Nisbet and Ambrose (1986), no fossils have been found in the barite except one poorly preserved shark tooth (Fig. 2). Other than fish teeth, two shark vertebrae and numerous dermal ossicles, the only fossils found so far in the associated sediments are minute valves of a discinid brachiopod. Most of the teeth were found in the basal part of the pebbly sandstone bed in the eastern part of the outcrop. These sediments are obscured by talus and vegetation in the southwestern part of the outcrop. The teeth are mostly small (less than 15 mm height); the largest tooth is approximately 70 mm high. Preservation in the hard, barite-rich matrix is generally poor to good, and while some teeth preserve delicate tips and cusplets, many teeth appear to lack their roots. The roots of the teeth appear in some cases to be replaced by barite making it difficult to distinguish them from the barite-rich matrix. Some of the teeth have chipped or worn apices, but because some teeth preserve delicate cusplets they were likely not transported far, if at all, despite their occurrence in a possible turbidite deposit. Most rock samples containing teeth also yield minute dermal ossicles.

The paleoenvironment of the lower unit of the Crescent Formation has been interpreted as open sea, based on planktic foraminiferans, with water depths of probably more than 400 m (Rau 1964, 1966). This is supported by the fish taxa described herein and the presence of minute discinid brachiopods. The lack of wood fragments and other detritus from terrestrial plants supports a depositional environment in deep water, far away from any land mass, however, clasts within the turbidites in the lower Crescent Formation are apparently of continental origin (Cady 1975; Babcock et al. 1992; McCrory and Wilson 2013). Cady (1975) estimated the continental margin to have been approximately 900 km east of its present location during deposition of the Crescent Formation. Phillips et al. (2017) and Ciborowski et al. (2020) hypothesized that Siletzia represents an ancient oceanic plateau, and McCrory and Wilson (2013) suggested the present-day Cocos and Carnegie oceanic ridges as possibly analogous to the paleodepositional setting for the lower Crescent Formation. Some parts of the lower Crescent Formation may be as old as 56 Ma (Babcock et al. 1994) however more recent age estimates for the lower member indicate that these strata are Ypresian, early Eocene, approximately 53–48 Ma (e.g., Wells et al. 2014; Eddy et al. 2017; Ciborowski et al. 2020).

## Material and methods

The locality is very difficult to access because the terrain is forested and very steep; Moen (1964) stated that he was unable to find the outcrop. Although only approximately 350 m north of the nearby road, the locality is also 300 m in elevation above the road. Rock samples were broken at the outcrop and those that contained teeth were brought back to the lab. Preparation of individual teeth in the hard matrix was done with a small pneumatic scribe and binocular microscope. A circular saw with a diamond blade was used to trim samples, along with tile nippers and similar hand tools. Some rock samples contain several teeth, and some of these were purposely not trimmed to preserve the original context. Once in the lab, all surfaces of the samples were examined with a binocular microscope and the best results were achieved at 30× magnification to find small teeth and brachiopod fossils. Approximately 210 teeth and numerous placoid scales were collected. Most of the fossils recovered are curated at the Burke Museum of Natural History and Culture, University of Washington, Seattle, Washington 98195 (UWBM—IP and VP are abbreviations for Invertebrate and Vertebrate Paleontology); three microscopic brachiopod fossils and several teeth and dermal ossicles found in processing of bulk rock samples are curated in the Swedish Museum of Natural History, Department of Palaeobiology, 10405 Stockholm, Sweden (NRM PAL). Precise locality details cannot be published herein due to United States rules protecting paleontological resources on federal land, however, this information is available and on file at the UWBM and NRM.

## Systematic paleontology

*Remarks*. We follow here mainly the systematic arrangement of Cappetta (2012) using Neoselachii sensu Compagno (1970) for modern sharks, rays, and skates instead of Elasmobranchii sensu Maisey (2012).

Stone and Shimada (2019) re-examined the phylogeny of extant odontaspidids and demonstrated that this group is not monophyletic and consequently resurrected the family Carchariidae. These authors suggested to restrict Odontaspididae to *Odontaspis*, while transferring *Carcharias* to Carchariidae. However, they limited these two families to extant taxa only not providing any information about the exact familial placements of the many fossil odontaspidids. Consequently, we use the systematic framework of Cappetta (2012) for Odontaspididae well knowing that this is rather artificial. A revision of fossil odontaspidids is beyond the scope of this study. We have cited references to taxonomic synonymies that have been recently published and are easily available. Lengthy synonymies for other taxa are outside the scope of this paper.

Class **Chondrichthyes** Huxley, 1880

Subclass **Elasmobranchii** Bonaparte, 1838

Cohort **Euselachii** Hay, 1902

Subcohort **Neoselachii** Compagno, 1970

Superorder **Squalomorphii** Compagno, 1973

Order **Hexanchiformes** de Buen, 1926

Family **Chlamydoselachidae** Garman, 1884

Genus ***Chlamydoselachus*** Garman, 1884

*Type species*. *Chlamydoselachus anguineus* Garman 1884, Recent.

*Stratigraphic range*. Campanian (Late Cretaceous) to Recent.

aff. ***Chlamydoselachus*** sp.

Figure 3a–c

**Fig. 3:**
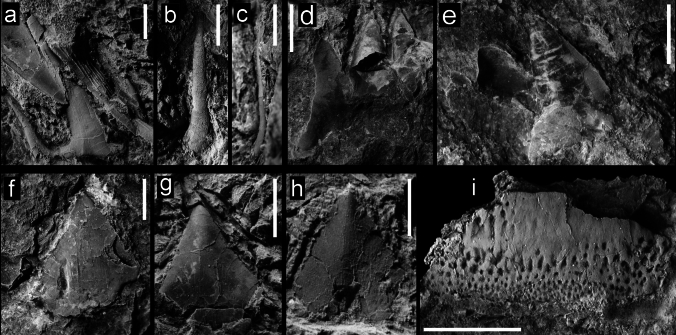
Teeth of Hexanchiformes, Squaliformes, and Heterodontiformes from the Hamma Hamma locality, lower unit of the Crescent Formation, early Eocene: **a** aff. *Chlamydoselachus* sp., UWBMVP 124381, complete tooth, labial view, with cusplet on the right side partially concealed by overlying tooth; **b** aff. *Chlamydoselachus* sp., UWBMVP 124382, isolated cusp with slight posteriorly directed bend near base; **c** aff. *Chlamydoselachus* sp., same specimen as **b** lateral view; **d** *Notorynchus* sp., UWBMVP 124383, lower lateral tooth, posterior half in lingual view; **e** *Notorynchus* sp., UWBMVP 124383, counterpart of **d** anterior half of tooth, labial view, with serrate cutting edge visible on right side; **f** *Isistius* cf. *trituratus*, UWBMVP 124384; **g** *Isistius* cf. *trituratus*, UWBMVP 124385; **h** *Isistius* cf. *trituratus*, UWBMVP 124386; **i** *Heterodontus* cf. *pineti*, NRM PAL P19790, lateral tooth. Scale bars equal 2 mm

*Material*. One tooth, UWBMVP 124381, and one isolated cusp, UWBMVP 124382.

*Locality*. “Maple Creek prospect”, lower unit of the Crescent Formation, Hamma Hamma River, Mason County, Washington (UWBM VP loc. C3331; UWBM IP loc. B9564).

*Description*. The incomplete tooth (Fig. 3a) is embedded in hard matrix and somewhat overlain by two other elasmobranch tooth crowns so that only the labial face is partly exposed. The central cusp and both lateral cusplets are preserved, one cusplet partly concealed by an appressed lamniform tooth. The main cusp is rather narrow with almost parallel cutting edges on the exposed portion. The labial face is rather flat transversely and completely smooth. Basally, it is rather stout and seemingly depressed rather than being circular. The lateral cutting edges reach the base of the cusp and extend at least for a short distance on the lateral heel between cusp and cusplets.

The lateral cusplets are well separated and divergent from the main cusp, and sigmoidal in labial view giving the crown a lyre-like appearance. The cutting edges are visible. The root is not preserved.

The second specimen tentatively assigned to aff. *Chlamydoselachus* sp. (Fig. 3b, c), an isolated cusp, has a smooth lingual face. The cusp is extremely long, thin, only slightly sigmoidal, and slightly bent posteriorly near the base.

*Remarks*. The dentition of both extant species, *Chlamydoselachus anguineus* and *C. africana* (Ebert and Compagno, 2009), is characterized by very conspicuous teeth, which are arranged in distinct files forming a clutching-type dentition sensu Cappetta (2012). The morphological differences in teeth of both extant species are not well established but are seemingly very minor. In the fossil record, conversely, teeth generally assigned to *Chlamydoselachus* show high degrees of morphological variation. This led Cappetta et al. (2021) to recognize three distinct morphotypes representing three different taxa: *Chlamydoselachus*, *Rolfodon* Cappetta et al. 2021, and *Dykeius* Cappetta et al. 2021. Teeth of late Cretaceous to late Miocene species currently assigned to *Rolfodon* (Cappetta et al. 2021) most conspicuously differ from those of *Chlamydoselachus* and *Dykeius* in the morphology of the root, i.e., a massive root that is longer than broad and has a pair of labio-lingual crests.

Teeth of *Dykeius garethi* Cappetta et al. 2021, from late Cretaceous rocks of Hornby Island, Canada, are significantly larger than those of any other chlamydoselachid with gracile and high, completely smooth cusps and cusplets, but no accessory cusplets. The root is much broader than long and has two very well-developed, high and labio-lingually oriented lingual crests.

Unfortunately, the two fragmentary teeth from the Crescent Formation lack the root preventing unequivocal assignment to any of the three chlamydoselachid genera. Nevertheless, the combination of slender and completely smooth cusp and cusplets, very divergent and in labial view sigmoidal lateral cusplets suggest a species of *Chlamydoselachus* rather than *Rolfodon* or *Dykeius*. More material is necessary to confirm the genus, and the fossils are too incomplete to assign to any species.

The extant species, *Chlamydoselachus anguineus*, is very wide-ranging with a patchy distribution ranging from 20 to 1500 m depths, whereas *C*. *africana* is only known from the western coast of Africa from depths of 300 to 1400 m (e.g., Ebert and Compagno 2009). Most fossil species of *Chlamydoselachus* described so far occur in deep-water sediments (e.g., Cigala-Fulgosi et al. 2009; Cappetta et al. 2021; but see Consoli (2008), for a different interpretation). The occurrence of chlamydoselachid teeth in deep-water sediments of the Crescent Formation thus agrees well with the general assumption of chlamydoselachids being benthopelagic deep-sea sharks. Although *Chlamydoselachus* has been reported from late Eocene rocks of Karaginsky Island in the Bering Sea (Nazarkin and Malyshkina 2020), the tooth reported herein is tentatively the northernmost Paleogene record for the eastern North Pacific Basin.

Family **Hexanchidae** Gray, 1851

Genus ***Notorynchus*** Ayres, 1855

*Type species*. *Notorynchus maculatus* Ayres, 1855, Recent.

*Stratigraphic range*. Ypresian (Eocene) to Recent.

*Remarks*. Generally, the fossil range of *Notorynchus* is considered to extend back to the early Cretaceous (Hauterivian) (e.g., Cappetta 2012). However, Underwood et al. (2011a) consider most Cretaceous teeth assigned to *Notorynchus* to belong either to *Gladioserratus* Underwood et al. 2011a, or another distinct hexanchiform, a view supported by Argyriou et al. (2022).

***Notorynchus*** sp.

Figure 3d, e

*Material*. One tooth, broken through the central section and preserved on two pieces of rock, UWBMVP 124383.

*Locality*. “Maple Creek prospect”, lower unit of the Crescent Formation, Hamma Hamma River, Mason County, Washington (UWBM VP loc. C3331; UWBM IP loc. B9564).

*Description*. The specimen is a lower lateral tooth broken through its central section and now preserved in two pieces of rock. The principal cusp (acrocone) is preserved (Fig. 3e) in one piece of rock, with two following distal cusplets (conules) which decrease in size and posteriorly discernable as impressions of the cusps or cusplets preserved in the other piece of rock. The principal cusp and distal cusplets are rather massive with rounded apices, which are directed distally. The mesial cutting edge of the principal cusp bears marked serrations basally with recurved apices. Remnants of the root are not identifiable. The apices are blunt with the mesial cutting edge being longer than the distal one. One of the preserved cusps is basally broken and exhibits an oval to roundish cross section.

*Remarks*. Extant hexanchids include three genera (*Notorynchus*, *Heptranchias* Rafinesque, 1810, and *Hexanchus* Rafinesque, 1810) whose interrelationships are still not completely resolved (e.g., Naylor et al. 2012). While *Hexanchus* is characterized, inter alia, by six gills slits, *Heptranchias* and *Notorynchus* have seven gill slits. All three have typical, mesio-distally elongated, labio-lingually compressed and comb-like lower teeth with successively arranged cusps.

The Hamma Hamma specimen, although somewhat incomplete, can be unambiguously identified as hexanchid due to the mesio-distally, consecutively arranged cusps. The distally decreasing size of cusps identify it as either *Hexanchus* or *Notorynchus* rather than *Heptranchias*. The presence of marked, rather strong and seemingly apically recurved basal mesial serrations indicates that this tooth belongs to *Notorynchus* rather than *Hexanchus*. Teeth of *Notorynchus* are less mesio-distally elongated than teeth of *Hexanchus*, which is, nevertheless, not possible to establish for the the Hamma Hamma specimen. Teeth of early Paleogene species of *Notidanodon* Cappetta 1975, possess very enlarged mesial cusplets and teeth of the Eocene hexanchid *Weltonia* Ward, 1979, differ in the very enlarged anterior-most principal cusp.

Teeth of the Eocene hexanchid, *Notorynchus serratissimus* (Agassiz, 1843) differ from typical teeth of extant *Notorynchus* because they have a low and mesially rounded root outline in lateral view and very massive cusps with evenly mesial serrations, and Adolfssen and Ward (2015) accordingly suggested to include this species in *Gladioserratus*, a hexanchid that is very abundant in Cretaceous rocks. Additionally, the principal cusp and first distal cusplet are of equal size in lower lateral teeth of *N*. *serratissimus* and differ in this from the Hamma Hamma specimen.

The Hamma Hamma *Notorynchus* most closely resemble upper Eocene teeth of *N*. *kempi* Ward, 1979, from England and Miocene *N*. *primigenius* (Agassiz, 1835) from Switzerland in having rather coarse mesial serrations and distally inclined principal cusp and first distal cusplets with a clear size difference between both. Although it is most parsimonious to assume that the specimen from the Hamma Hamma locality represents *N*. *kempi*, we refrain from assigning it to any species because it is incomplete.

While teeth of *Notorynchus* are quite common in Oligocene and Miocene strata of North America (Cappetta 2012), they are very rare in Eocene deposits of North America and mainly known from the Chesapeake region (*N*. *serratissiumus*, Ypresian; Kent 1994) and South Carolina (*N*. cf. *kempi*, Priabonian; Cicimurri and Knight 2019). *Notorynchus borealus* (Jordan and Hannibal 1923; originally described as *Notidanion boreale*) was based on teeth from the late Eocene Quimper Formation in western Washington, however its taxonomic status is somewhat questionable because few specimens are known (Ward 1979). Unidentified species of *Notorynchus* have also been reported from latest Eocene to Oligocene strata in Oregon and Washington (e.g., Welton 1972; Moore 1984). The presence of *Notorynchus* in the Hamma Hamma locality is the earliest record from the Pacific Coast of North America.

Order **Squaliformes** Goodrich, 1909

Family **Dalatiidae** Gill, 1893

*Remarks*. Although Gray (1851) is generally considered the author of Dalatiidae, he actually used ‘Dalatiana’, which was modified by Gill (1893) into Dalatiina, placing it together with his *Somniosina* into the new family Dalatiidae.

Genus ***Isistius*** Gill, 1864

*Type species*. *Scymnus brasiliensis* Quoy and Gaimard, 1824, Recent.

*Stratigraphic range*. Thanetian (late Paleocene) to Recent.

***Isistius*** cf. ***trituratus*** (Winkler, 1874b)

Figure 3f–h

*Material*. Nine isolated tooth crowns; figured specimens UWBMVP 124384, 124385, 124386, and six referred specimens, UWBMVP 124387–124392.

*Locality*. “Maple Creek prospect”, lower unit of the Crescent Formation, Hamma Hamma River, Mason County, Washington (UWBM VP loc. C3331; UWBM IP loc. B9564).

*Description*. This species is represented by isolated and slightly damaged tooth crowns, all without any visible remnants of the root, embedded in hard matrix. All are strongly compressed labio-lingually and the shape of the crown is that of an isosceles triangle with smooth cutting edges. The exposed crown face is devoid of any ornamentation and is approximately as wide as high; ranging in size from 1 mm, with the largest being 6 mm. The very triangular shape of the crowns is most similar to that of lower teeth of the dalatiid cookie cutter shark *Isistius*.

*Remarks*. Extant species of *Isistius* are characterized by a strongly heterodont dentition with very small, upper grasping teeth and relatively large, triangular lower cutting-type teeth. The fossil record of *Isistius* is dominated by lower teeth, probably because upper teeth are more difficult to identify. Up to now, only a single upper tooth of *Isistius* has been reported, from Eocene deposits of Europe (Adnet, 2006). All fossil lower teeth are generally assigned to two distinct species with different stratigraphic ranges: *I. trituratus* from the Paleocene to Eocene of Europe, North America and North Africa and *I. triangulus* (Probst, 1879) from the Miocene to Pliocene of Europe and South America (see Cappetta 2012, and references therein). Morphological differences between both species are feeble, but the teeth of *I. trituratus* can easily be differentiated from those of *I. triangulus* by having smooth cutting edges. So far, *Isistius* has not been reported from Oligocene or Pleistocene deposits (Kriwet and Klug 2009; Cappetta 2012). Although *I. trituratus* might have been common in eastern North America during the Eocene (e.g., Ward and Wiest 1990; Kent 1999), its exact distribution remains somewhat ambiguous. Welton (1974) reported *I*. cf. *trituratus* from Paleocene rocks in California, however the specimens from the Crescent Formation are the first Eocene occurrence of *Isistius* from the North Pacific Basin. Nevertheless, more and better-preserved material is needed to unambiguously confirm the identity.

Extant and extinct squaliform (dogfish) sharks are a highly diverse group with many taxa occurring in coastal and oceanic, cool temperate, and deep tropical waters worldwide and include some of the largest predators in the deep sea (Gartner et al. 1997; Compagno 1999; Musick et al. 2004). Most squaliforms are benthonic, but mesopelagic forms such as *Isistius* undertake nightly vertical migrations towards the sea surface in search of food (Martin and Treberg 2002; Adnet et al. 2021). The two extant species, *I. brasiliensis* (Quoy and Gaimard, 1824) and *I. plutodus* (Garrick and Springer, 1964), however, display very distinct depth distributions. *Isistius brasiliensis* inhabits depths of more than 3500 m, while *I. plutodus* generally occurs between 60 and 200 m water depth but was also reported from depths greater than 6000 m (e.g., Kiraly et al. 2003; Compagno et al. 2005). The occurrence of *I.* cf. *trituratus* thus agrees well with the inferred paleodepth of more than 400 m for deposition of the lower unit of the Crescent Formation.

Superorder **Galeomorphii** Compagno, 1973

Order **Heterodontiformes** Berg, 1937

Family **Heterodontidae** Gray, 1851

Genus ***Heterodontus*** Blainville, 1816

*Type species*. *Squalus philippi* Schneider, 1801, original designation, in Bloch and Schneider (1801) = *Heterodontus portusjacksoni* (Meyer, 1793). Recent.

*Stratigraphic range*. Valanginian (Early Cretaceous) to Recent.

***Heterodontus*** cf. ***pineti*** Case, 1981

Figure 3i

*Material*. A single, isolated tooth crown, NRM PAL P19790.

*Locality*. “Maple Creek prospect”, lower unit of the Crescent Formation, Hamma Hamma River, Mason County, Washington (UWBM VP loc. C3331; UWBM IP loc. B9564).

*Description*. The specimen is a lateral tooth embedded in rock so that only the crown is exposed. It is poorly preserved with most of its lingual margin missing, while the preserved part is slightly domed and displays faintly rugose ornamentation. Both labial and lingual crown faces are separated by a blunt, mesio-distally directed elevation, which is devoid of any distinct crest or cutting edge. This, however, might relate to the incompleteness of the crown. The apical elevation is displaced labially giving the tooth crown an asymmetric and bulbous appearance.

The lingual crown face is narrower and steeper, forming almost a transversally running depression. It possesses a distinct shallow pit-like ornamentation with pits that are smaller, more circular and densely arranged along the labial margin, whereas they become more sparsely arranged and labio-lingually elongated towards the apical elevation. The lingual ornamentation, as far as preserved, also comprises shallow, but more labio-lingually elongated and more parallel arranged pits.

*Remarks*. Two distinct dental morphological types can be distinguished in *Heterodontus* (e.g., Herman et al. 1993; Cappetta 2012; Hovestadt 2018). According to Hovestadt (2018), distinction between the species within a morphogroup is difficult, however, all fossil species that he included in *Heterodontus* can also be confidently assigned to one of these two groups.

Up to now, seven species have been described and figured from Eocene deposits that are considered valid (Hovestadt 2018). According to Hovestadt (2018), the species, *H*. *eocenicus* Tate, 1894, *H*. *pineti* Case, 1981, *H*. *wardenensis* Casier, 1966, and *H*. *woodwardi* Casier, 1946, display dental traits that are characteristic for the Morphotype 1 group, whereas *H*. *elongatus* Case and Borodin, 2000, *H*. *sowasheense* Case, 1994, and *H*. *vincenti* Leriche, 1905, should be assigned to Morphotype 2 group. The shape and ornamentation pattern of the single preserved lateral tooth crown described here associate it with extant members of the *Heterodontus portusjacksoni* group (Morphotype 1 group). The rather incomplete nature of the specimen, unfortunately, renders a definite identification difficult, but the bulbous crown and distinct ornamentation pattern ("conchoidal" ornamentation pattern of Case (1981)) resemble most closely what is seen in *H*. *pineti*. Lateral teeth of *H*. *eocenicus* and *H*. *woodwardi* are, conversely, flatter and the ornamentation patterns include reticulated costules (the lateral teeth of *H*. *wardenensis* are unknown). Similar lateral teeth of Morphotype 2 group likewise are flatter and display a different ornamentation pattern. Nevertheless, more and better-preserved specimens are necessary for an unambiguous taxonomic identification.

Case (1981) erected the species *H. pineti* for two teeth (one anterior, one lateral) from Priabonian strata in Georgia (USA) that are generally like those of *H*. *lerichei* Casier, 1943. While Parmley et al. (2003:158) judged the material to be ambiguous and Müller (1999) assumed that *H*. *pineti* might be a junior synonym of *H*. *janefirdae* Case, 1980 (most likely from Oligocene rather than Miocene sediments according to Müller 1999), Hovestadt (2018) deemed the morphological differences to be sufficient for identifying a distinct species, *H*. *pineti*. We follow this opinion here.

So far, *H*. *pineti* was only known from upper Eocene strata of Georgia and Louisiana in southeastern North America (Case 1981; Manning and Standhardt 1986). The new record reported here would expand the stratigraphic range to the early Eocene and the geographic range to northwestern North America.

Extant bullhead sharks (family Heterodontidae) are strictly bottom-dwellers on continental shelves and slopes up to ca. 300 m depth, but mostly associated with reefs and nearshore, shallow-water environments (Compagno 2001; Ebert et al. 2013). The record of the single, damaged tooth in the lower unit of the Crescent Formation likely represents an allochthonous rather than an autochthonous faunal element.

Order **Lamniformes** Berg, 1937

*Remarks*. Lamniform sharks represent the most abundant and diverse group of elasmobranchs in the Hamma Hamma assemblage including bathydemersal and epipelagic taxa which are characteristic of an open marine environment with considerable water depths. Most common are isolated tooth crowns that either represent members of carchariid or odontaspidid sandtiger sharks. The material, however, is too fragmentary and still embedded in hard matrix so that distinguishable characters in many cases cannot be identified conclusively for appropriate taxonomic assignments.

Family **Mitsukurinidae** Jordan, 1898

Genus ***Mitsukurina*** Jordan, 1898

*Type species*. *Mitsukurina owstoni* Jordan, 1898, Recent.

*Stratigraphic range*. Ypresian (early Eocene) to Recent.

***Mitsukurina*** cf. ***maslinensis*** (Pledge, 1967).

Figure 4a–d

**Fig. 4:**
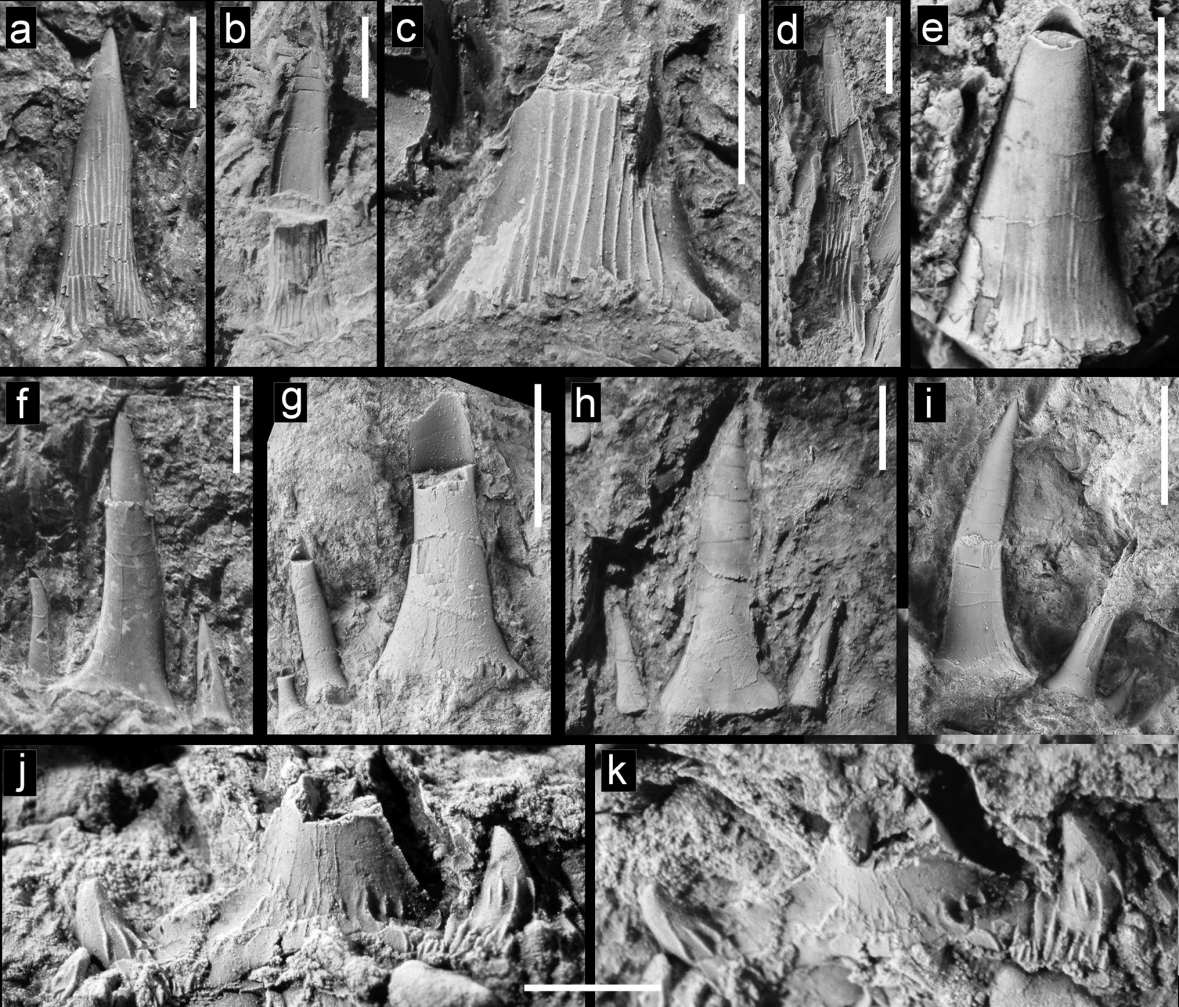
Teeth of Lamniformes (*Mitsukurina*, *Striatolamia, Odontaspis*, *Palaeohypotodus*), from the Hamma Hamma locality, lower unit of the Crescent Formation, early Eocene: **a** *Mitsukurina* cf. *maslinensis*, UWBMVP 124393, tooth crown in lingual view; **b** *M*. cf. *maslinensis*, UWBMVP 124394, tooth crown in labial view, part of tooth broken away showing mold with folds on lingual surface; **c** *M*. cf. *maslinensis*, UWBMVP 124395, base of tooth crown in lingual view; **d** *M*. cf. *maslinensis*, UWBMVP 124396, tooth crown in lingual view, partly concealing tooth of aff. *Chlamydoselachus* sp. in Fig. 3a; **e** *Striatolamia macrota*, UWBMVP 124401, tooth crown, lingual view; **f** *Odontaspis* cf. *winkleri*, UWBMVP 124402, lingual view; **g** *O*. cf. *winkleri*, NRM PAL P19791, lingual view; **h** *O*. cf. *winkleri*, UWBMVP 124403, labial view; **i** *O*. cf. *winkleri*, NRM PAL P19792, lingual view; **j** *Palaeohypotodus* sp., UWBMVP 124409, lingual view; **k** same as **j** apical view. Scale bars equal 2 mm

*Material*. Eight incomplete tooth crowns; four figured specimens, UWBMVP 124393–124396, and four referred specimens, UWBMVP 124397–124400.

*Locality*. “Maple Creek prospect”, lower unit of the Crescent Formation, Hamma Hamma River, Mason County, Washington (UWBM VP loc. C3331; UWBM IP loc. B9564).

*Description*. Tooth crowns without any remnants of the root or additional lateral cusplets are very small, very slender and slightly sigmoidal in lateral view. The labial face is convex mesio-distally, and devoid of any ornamentation. The lingual face, as far as can be ascertained, except for a thin band on either side next to the cutting edge is covered by dense enameloid folds that originate at the base, where they are approximately parallel and extend for 75 to 80 percent of cusp height. The crown of the most complete specimen (Fig. 4a; UWBMVP 124393) has its apex slightly twisted axially with respect to the base. A few other teeth also possess this slight axial twist toward the apex which makes it possible to identify them even when just the smooth labial face is exposed. The lateral cutting edges are weak and seemingly do not reach the base of the tooth.

*Remarks*. The extant goblin shark, *Mitsukurina owstoni*, has a wide distribution in both Atlantic and Pacific oceans, but is apparently absent in the Mediterranean Sea as well as polar regions (Ebert et al. 2021). The fossil record of *Mitsukurina* includes only two species, *M. maslinensis* (Pledge, 1967) and *M. lineata* (Probst, 1879) from the Paleogene of Australia and Neogene of Europe, respectively. Paleogene records are very rare in comparison to Neogene occurrences (e.g., Cappetta 2012, and references therein).

Teeth of *M. maslinensis* differ from those of *M. lineata* predominantly in the smaller size and the occasional presence of diminutive lateral cusplets in anterior teeth. The teeth from the Crescent Formation are very incomplete and do not allow observation of taxonomically important characters such as the possible presence of vestigial lateral cusplets. Nevertheless, we identify them as *M.* cf. *maslinensis*, representing anterior teeth, pending the discovery of more complete teeth. They differ from teeth of *Anomotodon* Arambourg, 1952, in lacking the medio-basal crest on the labial face (e.g., Carlsen and Cuny 2014) and stronger striations. More and better-preserved material is necessary to unambiguously verify the taxonomic assignment of the teeth from the Crescent Formation. These are, to our knowledge, the first record of *Mitsukurina* in Paleogene rocks of the North Pacific Basin.

Family **Odontaspididae** Müller and Henle, 1839

Genus ***Striatolamia*** Glikman, 1964

*Type species*. *Otodus macrotus* Agassiz, 1838, Eocene, Paris Basin (without more precise information).

*Stratigraphic range*. Danian (early Paleocene) to Priabonian (late Eocene).

*Remarks*. The systematic position of *Striatolamia* is still debated because of dental similarities to living and extinct carchariid and odontaspidid sand tiger sharks from which they nevertheless can be easily distinguished; Kriwet et al. 2016, provided a review of differentiating characters. Long (1992) and Purdy (1998) for example, dismissed *Striatolamia* and assumed it to be congeneric with *Carcharias* Rafinesque, 1810. Siversson (1995), Cappetta and Nolf (2005), and Cappetta (2012) conversely, regarded *Striatolamia* as a member of Mitsukurinidae, because of similarities to teeth of *Anomotodon hermani* Siversson 1992. *Striatolamia* has also been classified as a mitsukurinid recently by others, although mostly without further comment (e.g., Ebersole et al. 2019; Kolvalchuk et al. 2023; Popov and Lopyrev 2023), and still by some as an odontaspidid (e.g., Adnet 2006; Malyshkina 2021; Rodriguez et al. 2023; Verma 2023). We follow the more traditional view here and consider *Striatolamia* to be a valid taxon within Odontaspididae for reasons provided by Kriwet et al. (2016). A complete review and analysis of the exact taxonomic relationship of *Striatolamia* is beyond the scope of this paper.

***Striatolamia macrota*** (Agassiz, 1838).

Figure 4e

*Material*. One fragmentary tooth crown, UWBMVP 124401.

*Locality*. “Maple Creek prospect”, lower unit of the Crescent Formation, Hamma Hamma River, Mason County, Washington (UWBM VP loc. C3331; UWBM IP loc. B9564).

*Description*. Small very fragmentary main cusp like those assigned to *Mitsukurina* cf. *maslinensis* above but differs in that it is broader and more triangular with lingual enameloid folds that do not reach as far toward the apex. Additionally, the lingual crown face is not regularly convex mesio-distally but is medially flattened. The cutting edges are very prominent.

*Remarks*. We follow Ebersole et al. (2019) in considering *Striatolamia striata* (Winkler, 1874a) to be a junior synonym of *S. macrota* (Agassiz, 1838). Kovalchuk et al. (2023) and Verma (2023) have provided recent, lengthy, and comprehensive synonymies for *S*. *macrota*. The presence of short lingual apico-basally oriented folds that do not extend to the apex and the medially flattening of the lingual cusp face allow assignment of this fragmentary tooth to the extinct sandtiger shark *Striatolamia macrota*.

The dental similarities of *Striatolamia* with *Carcharias*, but also with *Mitsukurina* suggest that *Striatolamia* probably occupied similar ecological niches. *Striatolamia macrota* has been reported from early to late Eocene rocks in Washington, Oregon, California, Baja California Sur (Mexico) and Chiapas (Mexico) on the Pacific Coast of North America (e.g., Applegate 1968; Squires et al. 1992; Ferrusquía-Villafranca et al. 1999; Squires 2001). *Striatolamia* is a common element of pelagic elasmobranch faunas in the Eocene worldwide including very high latitudes (e.g., Welton and Zinsmeister 1980; Padilla et al. 2014) indicating that this shark genus was very successful. The reasons for its disappearance at the end of the Eocene, however, remain unknown.

Genus ***Odontaspis*** Agassiz, 1838

*Type species*. *Squalus ferox* Risso, 1810, Recent.

*Stratigraphic range*. Ypresian (early Eocene) to Recent.

***Odontaspis*** cf. ***winkleri*** Leriche, 1905

Figure 4f–i

*Material*. Nine incomplete teeth, some with remnants of the root; four figured specimens, NRM PAL P19791, 19792, UWBMVP 124402, 124403, and five referred specimens, UWBMVP 124404–124408.

*Locality*. “Maple Creek prospect”, lower unit of the Crescent Formation, Hamma Hamma River, Mason County, Washington (UWBM VP loc. C3331; UWBM IP loc. B9564).

*Description*. In addition to numerous isolated tooth cusps that most likely represent either carchariids or odontaspidids, some teeth are better preserved with lateral cusplets and remnants of the root. In anterior teeth, the main cusp is rather high and narrow, but flares basally. The preserved lateral cusplets are needle-like and either slightly divergent (in anterior teeth) or upright (in lateral teeth), decreasing in size mesially and distally. The lingual crown face is smooth, devoid of ornamentation. Anterior teeth additionally possess an awl-like, slender main cusp. In some specimens, a labio-basal short vertical ridge is present. The labial face is flat while the lingual face is slightly more convex mesio-distally. The cutting edges do not reach the base of the main cusp. Lateral teeth differ in having more slender and upright main cusps. The root lobes, as far as they are preserved display the typical morphology and shape for odonatspidids.

*Remarks*. Kovalchuk et al. (2023) provided a lengthy and comprehensive synonymy of this species. The preserved characters do not allow an unambiguous assignment to any species. However, they resemble teeth of *Odontaspis winkleri*, which was originally described from Lutetian strata of Belgium (Leriche 1905) but also occurs in Paleocene strata (e.g., Ward and Wiest 1990; Reinecke and Engelhard 1997) to some extent. However, typical teeth of *O. winkleri* differ in having more delicate lateral cusplets with more flared bases, which are much shorter compared to the height of the main cusp (compare Hovestadt and Steurbaut 2023). *Odontaspis winkleri* is very common in rocks deposited in pelagic environments of the Northern Hemisphere during the Ypresian, including North America, but also is known from the Southern Hemisphere (e.g., Smith et al. 1999; Kriwet 2005a; Rodriguez et al. 2023). Ward and Wiest (1990) reported *O. winkleri* from Danian and Thanetian (Paleocene) and Ypresian (Eocene) strata of Maryland and Virginia.

Case and Borodin (2000) introduced a new species, *O. carolinensis* from the Lutetian (middle Eocene) of North Carolina based on four mostly fragmentary teeth that very much resemble those of *O. winkleri*. Burdon (2009: figs. 16, 17), nevertheless, argued that the morphology of these teeth is different from typical teeth of *O. winkleri* from Europe and therefore considers *O. carolinensis* valid. The teeth figured by Burdon (2009) differ from those described here in displaying a more lanceolate main cusp; we agree that *O*. *carolinensis* appears to be a valid taxon.

Recently, Pollerspöck and Straube (2017) introduced a new Miocene deep-water shark, *Pseudoapristurus*, from Germany, characterized by very high and slender, pointed lateral cusplets that are well-separated from the main cusp, which also is high and very slender. However, the teeth of *Pseudoapristurus* differ from those of *O*. *winkleri* in possessing a very characteristic baso-lingual, reticulated ornamentation with a main cusp having a very circular cross section basally.

The teeth of *O. winkleri* resemble those of the extant sandtiger shark, *O. ferox* (Risso, 1810) and *O. winkleri* probably had similar feeding and environmental adaptations. We therefore hypothesize that *O. winkleri* was most likely a benthopelagic shark which we also infer for the species from the Crescent Formation described here because of similar dental morphologies.

Genus ***Palaeohypotodus*** Glikman, 1964

*Type species*. *Odontaspis rutoti* Winkler, 1874a; middle Paleocene (Selandian), Belgium.

*Stratigraphic range*. Danian (early Paleocene) to Priabonian (late Eocene).

***Palaeohypotodus*** sp.

Figure 4j, k

*Material*. A single, very fragmentary tooth, UWBMVP 124409.

*Locality*. “Maple Creek prospect”, lower unit of the Crescent Formation, Hamma Hamma River, Mason County, Washington (UWBM VP loc. C3331; UWBM IP loc. B9564).

*Description*. A single, very fragmentary tooth displays a basally broad main cusp, of which the upper part, probably two-thirds, is missing. The main cusp is flanked by broad and low lateral cusplets that are basally divergent but with apices that are curved towards the main cusp. The base of the main cusp and cusplets bear strong, flexuous basal folds. Along the basal margin of the crown, additional, fine vertical wrinkles occur. The cutting edge of the main cusp as well as those of the lateral cusplets are very prominent and reach the corresponding basis. The labial face of the main cusp is rather flat while the lingual face is strongly convex mesio-distally.

*Remarks*. The few traits that are preserved and accessible for examination unambiguously allows referral to genus *Palaeohypotodus*. Ebersole et al. (2024) recently reviewed the taxonomy of *Palaeohypotodus* concluding that the species *P*. *rutoti* as currently understood represents a wastebasket taxon and this species should probably best restricted to occurrences from the Selandian. The distinct morphology of the single specimen described here indicates it may represent a hitherto unknown species of *Palaeohypotodus*. However, more and better-preserved material is necessary to identify this species beyond any doubt. Generally, species of *Palaeohypotodus* are considered to represent sharks adapted to cool waters (e.g., Kriwet et al. 2016). *Palaeohypotodus* ranges stratigraphically from the early Paleocene to the Ypresian and occurs in Antarctica (Long 1992; Kriwet et al. 2016), Europe (Leriche 1951; Gurr 1962; Casier 1967; Herman 1972; Ward 1980; Rayner et al. 2009; Iserbyt and De Schutter 2012; Cappetta 2012), Greenland (Bendix-Almgreen 1969), Africa (Herman 1973; Cappetta 2012), central Asia (Glikman 1964), South America (Otero and Soto-Acuna 2015; Rodriguez et al. 2023) and North America (Ward and Wiest 1990; Ebersole et al. 2024). The only previous record of *Palaeohypotodus* from the Pacific Coast of North America was a single tooth from the middle-early Eocene uppermost part of the Crescent Formation on the south side of the Olympic Peninsula (Squires and Goedert 1994). The stratigraphically youngest occurrences are from late Eocene rocks in the Southern Hemisphere suggesting that high latitude environments might have served as refugium before the extinction of *Palaeohypotodus* at the end of the Eocene.

Genus ***Brachycarcharias*** Cappetta and Nolf, 2005

*Type species*. *Lamna lerichei* Casier, 1946; lower Eocene, Ypresian, Forest-lez-Bruxelles, Belgium.

*Stratigraphic range*. Danian (early Paleocene) to Lutetian (middle Eocene).

***Brachycarcharias*** cf. ***lerichei*** (Casier, 1946).

Figure 5a–d

**Fig. 5:**
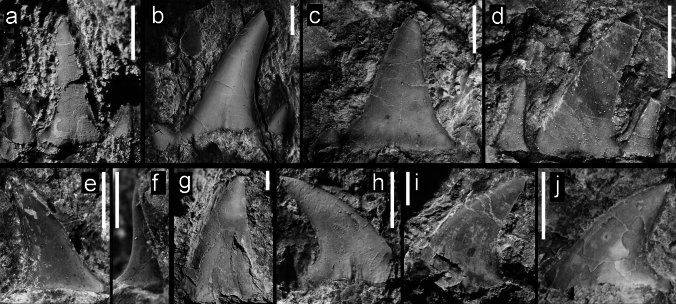
Teeth of Lamniformes (*Brachycarcharias*, *Macrorhizodus, Jaekelotodus*), from the Hamma Hamma locality, lower unit of the Crescent Formation, early Eocene: **a** *Brachycarcharias* cf. *lerichei*, UWBMVP 124410, labial view; **b** *B*. cf. *lerichei*, NRM PAL P19793, labial view; **c** *B*. cf. *lerichei*, UWBMVP 124411, labial view; **d** *B*. cf. *lerichei*, UWBMVP 124412, labial view; **e** *Macrorhizodus praecursor*, UWBMVP 124415, lingual view; **f** *M*. *praecursor*, same specimen as **e**, lateral view; **g** *M*. *praecursor*, UWBMVP 124416, labial view; **h** *Jaekelotodus* sp., UWBMVP 124417, labial view; **i** *Jaekelotodus* sp., UWBMVP 124418, labial view; **j** *Jaekelotodus* sp., UWBMVP 124419, lingual view. Scale bars equal 2 mm

*Material*. Three incomplete anterior and three incomplete lateral teeth; four figured specimens NRM PAL P19793, UWBMVP 124410, 124411, 124412, and two referred specimens UWBMVP 124413, 124414.

*Locality*. “Maple Creek prospect”, lower unit of the Crescent Formation, Hamma Hamma River, Mason County, Washington (UWBM VP loc. C3331; UWBM IP loc. B9564).

*Description*. Three incomplete teeth preserve an upright to slightly distally inclined main cusp with a single pair of lateral cusplets in labial view. Unfortunately, the teeth lack roots. The labial face of the main cusp is flat and completely devoid of any ornamentation, but bears a short, vertical ridge basally. The cutting edges of the main cusp reach the base. The lateral cusplets are broadly triangular, rather low and somewhat divergent. The mesial cusplet is more slender with almost straight cutting edges, while the distal cusplet is broader with a concave mesial cutting edge. Both also lack any ornamentation.

Lateral teeth display a more triangular, comparably lower main cusp. Unfortunately, these lack any remnants of the root and are exposed in labial view only. The preserved lateral cusplets are low, triangular and not well separated from the main cusp. Both main cusp and lateral cusplets are devoid of ornamentation.

*Remarks*. Based on the preserved characters, the teeth are assigned to the extinct sandtiger shark *Brachycarcharias* and are most similar to those of the type species, *B*. *lerichei* which is known from Ypresian deposits of Europe, North America, Africa, and Antarctica (e.g., Cappetta 2006). Unfortunately, the teeth from the Hamma Hamma locality are too incomplete to unambiguously assign them to any species. The specimen in Fig. 5d displays rather high lateral cusplets and its assignment to *B. lerichei* is probably questionable as it could represent *B. atlasi* (J.A. Ebersole, pers. comm. 2024). However, it would be necessary to also study the lingual crown face to unambiguously assign it to *B. atlasi*, which currently is not possible. Consequently, we refer this tooth tentatively to *B. lerichei* pending more and better-preserved material. *Brachycarcharias* was most likely an epipelagic shark, which was adapted to warmer waters and its disappearance (like that of *Striatolamia*) at the end of the Eocene might be related to the gradual cooling of sea-water temperatures and the establishment of vast polar ice shields at the Eocene–Oligocene transition (e.g., Marramà et al. 2018).

Genus ***Jaekelotodus*** Menner, 1928

*Type species*. *Hypotodus trigonalis* Jaeckel, 1895; from the lower Oligocene, Ukraine.

*Stratigraphic range*. Danian (early Palaeocene) to Rupelian (early Oligocene).

***Jaekelotodus*** sp.

Figure 5h–j

*Material*. Seven incomplete teeth; three figured specimens, UWBMVP 124417–124419, and four referred specimens: UWBMVP 124423, 124424, 124425, and 124427. Two additional specimens, 124421 and 124422 are not well exposed but are tentatively referred here as well.

*Locality*. “Maple Creek prospect”, lower unit of the Crescent Formation, Hamma Hamma River, Mason County, Washington (UWBM VP loc. C3331; UWBM IP loc. B9564).

*Description*. Only isolated tooth crowns, lacking the root and lateral cusplets, are preserved. They display either the labial (Fig. 5h, i) or lingual crown face (Fig. 5j). All specimens display a rather distally hooked crown. The labial face is flat without any ornamentation but may display a short basal vertical groove. The lingual face is rather convex transversely and also completely devoid of any ornamentation. The cutting edges reach towards the base of the crown in all specimens.

*Remarks*. Unfortunately, these teeth are too incomplete for any definite taxonomic assignment. The distally hooked cusp with seemingly complete cutting edges and completely smooth labial and lingual crown faces allow identification of them as upper lateral teeth of *Jaekelotodus*, probably of *J*. *trigonalis* (see Cappetta and Nolf 2005). However, any of the other 14 species assigned to *Jaekelotodus* cannot be ruled out. In North America, reported records of *Jaekelotodus* are rare up to now. All North American material was either assigned to *J*. *trigonalis* from the Lutetian of the Chesapeak area (Ward and Wiest 1990) or *J*. *robustus* from the Thanetian–early Eocene of Mississippi (Case 1994) and Lutetian–Bartonian of Alabama (Ebersole et al. 2019).

The new record reported here from the Ypresian fall well within the known stratigraphic range of this odonatspidid in North America. Although very poorly preserved they are important because they extend the geographic distribution of this epipelagic shark into the North Pacific Basin and represent the northernmost Paleogene record on the North American continent.

Family **Lamnidae** Müller and Henle, 1838

Genus ***Macrorhizodus*** Glikman, 1964

*Type species*. *Oxyrhina falcata* Rogovich, 1861; from the upper Priabonian (Upper Eocene) of Ukraine.

*Stratigraphic range*. Ypresian (early Eocene) to Rupelian (early Oligocene).

***Macrorhizodus praecursor*** (Leriche, 1905).

Figures 2, 5e–g

*Material*. Five incomplete teeth; three figured specimens, UWBMVP 124415–124416, 124420; and two referred specimens, UWBMVP 124426 and 124428.

*Locality*. “Maple Creek prospect”, lower unit of the Crescent Formation, Hamma Hamma River, Mason County, Washington (UWBM VP loc. C3331; UWBM IP loc. B9564).

*Description*. Lamnid sharks are only represented by rare, incomplete teeth of *Macrorhizodus*, all still embedded in matrix. The crowns of antero-lateral teeth are high (the largest is approximately 26 mm long), rather narrow and faintly inclined distally with a very mesio-distally convex lingual face. The tooth crown of the specimen in Fig. 2, which also is accessible in lingual view, is embedded in barite and very weathered and crushed but displays the outline of an asymmetrical root with an elongated and quite narrow mesial root lobe and a shorter, broader distal root lobe with a rounded extremity. This specimen is an anterolateral tooth, as is the isolated tooth crown in Fig. 5g.

*Remarks*. Kovalchuk et al. (2023) provided a lengthy and comprehensive synonymy for this species. Teeth of *Macrorhizodus* differ from teeth of *Isurus* Rafinesque 1810, most significantly in having a massive cusp that is lingually inclined with rather slender and elongated root lobes in anterior teeth, a narrow lingual neck and a very prominent lingual root protuberance.

Five species of *Macrorhizodus* each with successive stratigraphic occurrences are currently recognized, which represent an evolutionary lineage according to Zhelezko and Kozlov (1999): *M*. *nolfi* Zhelezko and Kozlov, 1999, from the Ypresian, *M*. *praecursor* (Leriche, 1905) (= *M*. *americanus* (Leriche, 1942); Ebersole et al. 2019) from the Ypresian and Bartonian, *M*. *falcatus* (Rogovich, 1861) from the Priabonian, and *M*. *flandricus* (Leriche 1910) from the Rupelian. The two Eocene species, *M*. *nolfi* and *M*. *praecursor*, display distinct paleogeographic distributions with *M*. *nolfi* occurring in England, Kazahkstan and Denmark, whereas *M*. *praecursor* has a worldwide distribution during the middle to late Eocene (e.g., Adnet et al. 2021, and references therein). The main characters distinguishing both species are the presence of small and vestigial lateral cusplets or hump-like structures on the lateral heels in *M*. *nolfi*, which also is characterized by smaller overall size of the teeth. Although the specimens from the Hamma Hamma locality are incomplete, they seem to lack such vestigial lateral cusplets and therefore are assigned here to *M*. *praecursor*.

Family **Otodontidae** Glikman, 1964

*Remarks*. According to Cappetta (2012), Otodontidae is comprised of two genera of iconic macrophageous sharks, *Otodus* and *Parotodus*. He further separated the genus *Otodus* into three subgenera based on dental characters: *Otodus* (*Otodus*) Agassiz, 1838, *Otodus* (*Carcharocles*) (Jordan and Hannibal, 1923), and *Otodus* (*Megaselachus*) (Glikman, 1964). Recently there have been arguments in favor of suppressing the use of otodontid subgenera (e.g., Ehret and Ebersole 2014; Shimada et al. 2017; Boessenecker et al. 2019), whereas others continue to use the subgeneric assignments (e.g., Ebersole et al. 2019; Kovalchuck et al. 2023), and some (e.g., Perez et al. 2019) use them as genera; there still seems to be no consensus. We follow Cappetta (2012) herein. As noted by Brignon (2021), *Carcharocles* Jordan and Hannibal, introduced by them as a new genus in Jordan (1923), a classification of fishes, is often misattributed to the separate paper on fossil sharks (Jordan and Hannibal 1923) published in the same year.

Genus ***Otodus*** (***Carcharocles***) Jordan and Hannibal, in Jordan, 1923

*Type species*. *Squalus auriculatus* Blainville, 1818, from the Lutetian (middle Eocene) of Belgium.

*Stratigraphic range*. Ypresian (early Eocene) to Burdigalian (early Miocene).

***Otodus (Carcharocles) auriculatus*** (Blainville, 1818).

Figure 6a–e

**Fig. 6:**
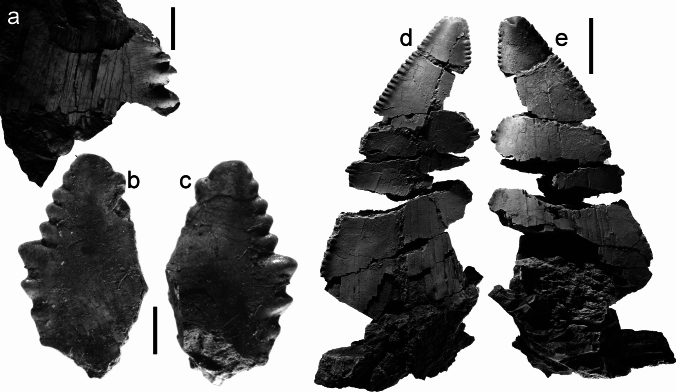
Teeth of Lamniformes (*Otodus*) from the Hamma Hamma locality, lower unit of the Crescent Formation, early Eocene: **a** *Otodus* (*Carcharocles*) *auriculatus*, UWBMVP 124429, tooth fragment showing irregular serrations; **b** *O*. (*C*.) *auriculatus*, UWBMVP 124430, left lateral cusplet, labial view; **c** same as **b** lingual view; **d** same specimen as in **b** main cusp, labial view; **e** same as **d** lingual view. Scale bars for **a**–**c** equal 2 mm; for **d**, **e** equal 10 mm

*Material*. A single incomplete and fragmentary tooth with one associated lateral cusplet, UWBMVP 124430, and a second tooth represented by a small fragment, UWBMVP 124429.

*Locality*. “Maple Creek prospect”, lower unit of the Crescent Formation, Hamma Hamma River, Mason County, Washington (UWBM VP loc. C3331; UWBM IP loc. B9564).

*Description*. This otodontid is represented by an incomplete and fragmentary tooth crown (Fig. 6d, e), which would have had a height exceeding 70 mm if complete, the largest tooth from the Hamma Hamma assemblage, as well as its now isolated lateral cusplet (Fig. 6b, c). The cusplet was originally part of the single tooth when found, but weathering had reduced most of that portion of the tooth to powder. The tooth crown is slender, high and distally curved with continuous, somewhat irregular serrated cutting edges. The small, serrated lateral cusplet possesses very irregular serrae. The tooth fragment (Fig. 6a) also displays irregular serrations.

*Remarks*. The tooth crown and lateral cusplet from the Hamma Hamma locality are very similar to those assigned to *Otodus* (*Otodus*) and *Otodus* (*Megaselachus*), but differ from species of the first subgenus by having serrated cutting edges, and from species of the second subgenus by the more irregular serrations along the tooth crown and on the lateral cusplet, as well as a much more developed lateral cusplet, which are much smaller in *Otodus* (*Megaselachus*), if present at all. Therefore, the Hamma Hamma tooth crown and lateral cusplet are assigned to *Otodus* (*Carcharocles*).

Identification of species within *Otodus* (*Carcharocles*) is generally based on serration patterns of the central cusp and the form and serration of lateral cusplets (Kriwet et al. 2016). Following Cappetta (2012), species generally assigned to *Otodus* (*Carcharocles*) are* O*. (*C*.) *aksuaticus* (Menner, 1928), *O*. (*C*.) *angustidens* (Agassiz, 1835), *O.* (*C*.) *auriculatus*, *O.* (*C*.) *poseidoni* Zhelezko in Zhelezko and Kozlov, 1999 (including at least three different “subspecies”), *O.* (*C*.) *sokolovi* (Jaekel, 1895), and *O*. (*C*.) *subserratus* (Agassiz, 1838).

The fossils from the Hamma Hamma locality are best assigned to *O.* (*C*.) *auriculatus* based on the slender crown with slightly irregular serrae, which diminish in size apically, and the ragged lateral cusplets. In *O.* (*C*.) *sokolovi*, conversely, the serration of the lateral cusplets is finer and more regular. *Otodus* (*C*.) *auriculatus* was almost globally distributed during most of the Eocene, and especially in many Atlantic and Tethyan open marine settings (e.g., Ward and Wiest 1990; Long 1992; Cappetta 2012; Carlsen and Cuny 2014; Ehret and Ebersole 2014). There is an early Eocene record of *O.* (*C*.) *auriculatus* from Chatham Island (Keyes 1987) in the southwestern Pacific Basin. From the North Pacific Basin the only previous records of *O.* (*C*.) *auriculatus* are from Japan (Yabumoto and Uyeno 1994) in the “Kattachi Formation”, now included in the Miike Formation of middle Eocene age (Iguchi 2016), the Namigata Formation (Tanaka et al. 2006) shown to be latest Eocene (Matsubara 2013), and middle Eocene rocks in southern Mexico (Ferrusquía-Villafranca et al. 1999). This is the first report of *O*. (*C*.) *auriculatus* from the northeastern Pacific Ocean.

Family **Alopiidae** Bonaparte, 1838

Genus ***Alopias*** Rafinesque, 1810

*Type species*. *Alopias macrourus* Rafinesque, 1810, Recent.

*Stratigraphic range*. Ypresian (early Eocene) to Recent.

***Alopias*** sp.

Figure 7a–c

**Fig. 7:**
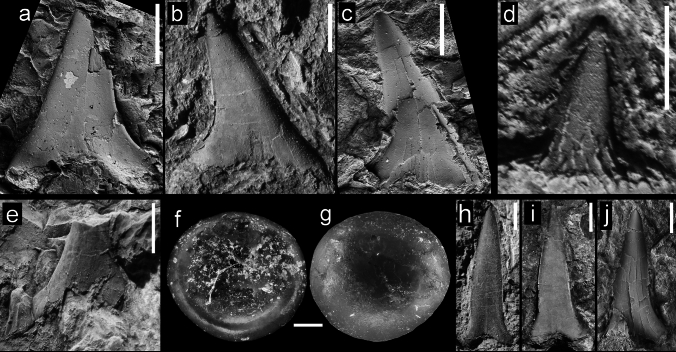
Teeth of Lamniformes (*Alopias*, Carchariidae and Odontaspididae incertae sedis), Carcharhiniformes, and Osteichthyes (*Egertonia*), from the Hamma Hamma locality, lower unit of the Crescent Formation, early Eocene: **a** *Alopias* sp., NRM PAL P19795, labial view; **b** *Alopias* sp., UWBMVP 124431, labial view; **c** *Alopias* sp., NRM PAL P19796, labial view; **d** Scyliorhinidae indeterminate, UWBMVP 124433, lingual view; **e** Carcharhiniformes indeterminate, UWBMVP 124434, labial(?) view; **f** *Egertonia* cf. *isodonta*, NRM PAL P19797, basal view; **g** same specimen as **f**, apical view; **h**–**j**, unidentified carchariids or odontaspidids, **h** UWBMVP 124435, labial view; **i** UWBMVP 124436, labial view; **j** NRM PAL P19794, labial view. Scale bars for **f**, **g** equal 0.2 mm, all others 2 mm

*Material*. Four teeth: a single nearly complete tooth with parts of the root preserved, UWBMVP 124431, and three isolated tooth crowns; figured specimens NRM PAL P19795, 19796, and referred specimen UWBMVP 124432.

*Locality*. “Maple Creek prospect”, lower unit of the Crescent Formation, Hamma Hamma River, Mason County, Washington (UWBM VP loc. C3331; UWBM IP loc. B9564).

*Description*. The nearly complete tooth assigned to *Alopias* (Fig. 7b; UWMBVP 124431) is embedded in matrix and only exposed in labial view. The cusp is rather slender, triangular, completely devoid of any ornamentation, and slightly inclined distally with an almost straight mesial cutting edge and a basally convex distal cutting edge, which straightens apically. The cutting edges extend to the base forming a very short distal heel-like structure. Lateral cusplets are absent. The basal margin of the crown is damaged so that its true form (straight or curved) remains ambiguous. The root is very incomplete and only the distal root lobe is discernable, which has a convexly curved outer margin. The root lobe does not extend far distally below the crown. The three isolated tooth crowns lack any remnants of the root and are fragmentary and very triangular with a flat and unornamented labial cusp face. The cutting edges extend to the base of the cusp and continue distally to form low heels.

*Remarks*. The tooth and the two tooth crowns from the Hamma Hamma locality resemble most closely teeth of the *Alopias superciliosus* (Lowe, 1841) group. In the Eocene, *Alopias* is predominantly known from tropical Tethyan realms of Europe and North Africa (Case and Cappetta 1990; Underwood et al. 2011b; Cappetta 2012; Adnet et al. 2021). Three species occur in the Eocene of Europe: *Alopias crochardi* Ward, 1978, from the Ypresian (early Eocene) of England, *A*. cf. *denticulatus* Cappetta, 1981, from the Ypresian (early Eocene) of France (Adnet 2006), which was originally described from Ypresian (early Eocene) rocks of Morocco. *Alopias leensis* Ward, 1978, from the middle-late Eocene boundary in England, was reassigned to *Usakias* Zhelezko and Kozlov 1999, by Kozlov (2000). Conversely, in North America, *Alopias* is very rare in Eocene strata. White (1956) ascribed some Eocene teeth from Alabama to a new species, *A. latidens alabamensis*. This species, however, is somewhat dubious and needs a detailed reanalysis to confirm its taxonomic status. For example, Ebersole et al. (2019) placed this species into synonymy with *Negaprion gilmorei* (White, 1956). Burdon (2009) presented some Middle Eocene (Lutetian) teeth assigned to *Alopias latidens* Leriche, 1909, from North Carolina, which slightly resemble the tooth described here. Nevertheless, we do not assign the tooth from the North Pacific Basin to any species until more material is available. This is the first Eocene record of *Alopias* from the North Pacific Basin.

Order **Carcharhiniformes** Compagno, 1973

Family **Scyliorhinidae** Gill, 1862

**Genus and species indeterminate**

Figure 7d

*Material*. A single incomplete tooth, UWBMVP 124433.

*Locality*. “Maple Creek prospect”, lower unit of the Crescent Formation, Hamma Hamma River, Mason County, Washington (UWBM VP loc. C3331; UWBM IP loc. B9564).

*Description*. The single small and incomplete tooth is exposed in lingual view based on the mesio-distally very convex cusp face. The cusp is cone-shaped with a rather blunt apex. The cutting edges seem weak extending to the base of the cusp. Lateral cusplets are not preserved, which, however, is more likely due to damage during or after deposition. There are well-developed short and flexuous ridges in the basal part of the cusp that extend apically for a short distance and basally across a narrow rim-like shelf. Some of the folds bifurcate basally. The basal margin of the crown is almost vertical in lateral view and the upper edge of the rim-like shelf appears very crenulated due to the bent ridges. The root is not preserved.

*Remarks*. The sturdy shape of the cusp and strong ornamentation suggests that this tooth belongs to a member of carcharhiniform sharks, most likely a scyliorhinid. However, more and better material showing both labial and lingual faces as well as potential lateral cusplets is necessary for any more definite taxonomic assignment.

Order **Carcharhiniformes** Compagno, 1973

**Family, genus and species indeterminate**

Figure 7e

*Material*. A single, very incomplete tooth, UWBMVP 124434.

*Locality*. “Maple Creek prospect”, lower unit of the Crescent Formation, Hamma Hamma River, Mason County, Washington (UWBM VP loc. C3331; UWBM IP loc. B9564).

*Description*. This very incomplete tooth displays a rather broad, basally flaring, upright main cusp in labial view, which is approximately symmetrical. The labial face is flat and devoid of any ornamentation. The upper part of the cusp is missing. The cutting edge extends to the base of the main cusp and is continuous with the one preserved lateral cusplet. This lateral cusplet is very small with a very distinct ornamentation pattern consisting of a ridge that follows the outline of the lateral cusplet. An additional ridge occurs connecting the main ridge with the cutting edge close to the transition of the cusp-cusplet cutting edge. Shorter ridges also are present. The base of the crown is heavily damaged and the root is missing as is the lateral cusplet of one side.

*Remarks*. It is not possible to assign this very fragmentary tooth to any taxon although the ornamentation pattern of the single preserved lateral cusplet is very distinct. Based on this the tooth might represent a scyliorhinid, but more and better-preserved material is necessary for any conclusive identification.

Class **Osteichthyes** Huxley, 1880

Subclass **Actinopterygii** sensu Goodrich, 1930 (see Schwarzhans et al., 2020).

Division **Teleosteomorpha** Arratia et al., 2004

Subdivision **Teleostei** Müller, 1845

Supercohort **Teleocephala** de Pinna, 1996

Cohort **Elopomorpha** Greenwood et al., 1966

Order **Elopiformes** Sauvage, 1875

Family **Phyllodontidae** Dartevelle and Casier, 1943

Subfamily **Phyllodontinae** Dartevelle and Casier, 1949

Genus ***Egertonia*** Cocchi, 1864

*Type species*. *Egertonia isodonta* Cocchi, 1864, Ypresian (early Eocene), Isle of Sheppey, United Kingdom.

*Stratigraphic range*. Maastrichtian (late Cretaceous) to Bartonian (middle Eocene).

***Egertonia*** cf. ***isodonta*** Cocchi, 1864

Figure 7f, g

*Material*. A single, isolated tooth, NRM PAL P19797.

*Locality*. “Maple Creek prospect”, lower unit of the Crescent Formation, Hamma Hamma River, Mason County, Washington (UWBM VP loc. C3331; UWBM IP loc. B9564).

*Description*. The single tooth is circular in outline with a very low, slightly convex crown. The crown surface and its base are completely smooth without any traces of ornamentation. The basal edge of the crown is broad. A very shallow and wide pulp cavity, which is not surrounded by a distinct band of dentine, is discernable in basal view.

*Remarks*. Several actinopterygian fishes with sub-circular to circular, hemispherical crushing teeth such as the pycnodontid *Pycnodus* Agassiz, 1835, the phyllodontids *Egertoni* Cocchi, 1864, and *Phyllodus* Agassiz, 1837 (non 1835; see Brown 1890), and the albulid *Albula* Gronow, 1763, co-occurred in Eocene marine environments. Complete dentitions of these taxa are easy to discriminate, but it is more problematic to separate them based on isolated teeth. Nevertheless, all taxa display characters that help in categorizing isolated teeth with a fair degree of confidence. Accordingly, teeth of *Pycnodus*, which is the last survivor of a previously very successful stem teleostean lineage (Kriwet 2005b), are not circular but rather semi-circular to oval in outline and display an ornamented occlusal crown surface when not abraded. Whereas teeth of *Paralbula* Blake, 1940, are very similar to the one presented here, especially in having unornamented, convex crowns, they differ in that they are more domed and have a central pulp cavity that is surrounded by a distinct band of dentine. Teeth of *Phyllodus* are circular (in lateral files) to oval (in median files) and thus might resemble that from Hamma Hamma locality in some cases. These teeth, nevertheless, can be distinguished by a less domed crown and presence of ornamentation patterns. Teeth of *Albula* can be readily differentiated from the tooth described here by a nearly flat to slightly convex occlusal surface and having a base with either straight and parallel, biconvex or tapering edges. Consequently, it is most plausible to assign the isolated tooth to the genus *Egertonia*.

This taxon currently is considered monospecific (Ebersole et al. 2019), although some other species previously were described: *E*. *gaultina* Cornuel, 1877, from the upper Gault (Lower Cretaceous) of France, which has been considered invalid and was synonymized with *Casierius heckelii* Estes, 1969a; *E. gosseleti* Leriche, 1900, from the lower Eocene of France, which represents a junior synonym of *E*. *isodonta*; *E*. *insignis* Cocchi, 1864, from the Paleocene of Belgium, which has to be considered invalid (nomen nudum); and *E*. *stromeri* Weiler, 1929, from the Eocene of Egypt, which was transferred to *Paralbula* by Estes (1969b).

While *Egertonia* is rather common in Paleogene, especially Eocene strata; Cretaceous records are less common and known unambiguously only from Maastrichtian strata in Madagascar (Ostrowski 2021) and India (Halliday et al. 2016). North American records from Campanian and Maastrichtian rocks (Wheeler 1966; Glaser 1979) need to be re-evaluated. Nonetheless, it is evident that this phyllodont teleost had a rather wide distribution already in the late Cretaceous that included the Southern Hemisphere, while this fish only occurred in the Northern Hemisphere during the Paleogene according to our current knowledge. During the Eocene, *Egertonia* was widespread across North America, having been found in Alabama, Georgia, Mississippi, North Dakota, South Carolina, and Virginia (Ebersole et al. 2019). The new record presented here expands the range of *Egertonia* into the northeastern Pacific Ocean during early Eocene time.

*Egertonia* was predominantly a marine, near-coastal fish that nevertheless also entered estuaries and even continental environments (e.g., Ebersole et al. 2019). The occurrence of the single tooth described here from the deep-water deposits of the Crescent Formation most likely represents an allochthonous element.

## Conclusions

The Hamma Hamma fish fauna includes at least 14 taxa and is the first early Eocene shark assemblage to be reported from western North America. It includes the first early Eocene records of aff. *Chlamydoselachus*, *Notorynchus*, *Isistius*, *Mitsukurina*, *Jaekelotodus*, *Otodus* (*C.*) *auriculatus*, *Alopias* and the teleost *Egertonia* from the North Pacific Basin. Most common are robust, indeterminate teeth (Fig. 7h–j, and 25 additional specimens, lot UWBMVP 124437) of carchariids and odontaspidids (sensu Stone and Shimada 2019), while remains of typical deep-sea sharks such as squaliforms, with the exception of one probably mesopelagic squaliform taxon, as well as batomorphs (rays and skates) have not yet been discovered. Elasmobranch teeth are common at the Hamma Hamma site, but unfortunately taxonomic identification is rendered difficult or even impossible by their incomplete and fragmentary nature. Many seem to lack the root, or the root cannot be readily differentiated from the enclosing matrix, whereas in some specimens remnants of the upper root portions are visible. Moreover, all specimens are embedded in extremely hard matrix, which does not allow extraction of the teeth without damaging them further. Importantly, the hard matrix does not allow for disaggregation and screening; that a tooth is discovered at all is partly serendipitous, depending on how each piece of rock is broken. In spite of this, very small teeth may actually be rare in this deposit because very small dermal denticles were often found, so it is unlikely that small teeth were missed because of collecting bias. However, this report on the Hamma Hamma assemblage should be considered preliminary because more detailed collecting over a longer time would undoubtedly reveal more taxa.

The extant goblin shark, *Mitsukurina owstoni* and frilled shark, *Chlamydoselachus anguineus* have a depth range of ca. 100 to more than 1000 m, but seem to regularly frequent deep-water seamounts (e.g., Compagno 2001; Kukuev and Pavlov 2008; Delgado et al. 2017), which is in good accordance with the depositional setting for the submarine basalts of the lower Crescent Formation interpreted as being, in part, ancient seamounts. The extant species, *Notorynchus cepedianus* (Péron, 1807) has a depth range of 0 to ca. 600 m (Cox and Francis 1997) but is seemingly very common inshore up to ca. 80 m depth (e.g., Compagno et al. 1989). Therefore, *Notorynchus* is considered a demersal shallow water hexanchiform that also thrives into the mesopelagic zone, but not deeper.

The clear dominance of lamniform sharks, especially of odontaspidids, is striking but most likely represents a collection or preservational artifact. This interpretation is supported by the complete lack of fossils of batoids (rays and skates). The two unidentified small carcharhiniforms may represent hitherto unknown Ypresian taxa. The taxonomic composition and dominance of epipelagic lamniform sharks indicate that this is a taxonomically highly uneven association with many typical deep-water taxa such as typical squaliforms, and rajiforms still to be discovered. So far, only one type of bony fish (*Egertonia*) has been recovered, which may be allochthonous. The Hamma Hamma assemblage nevertheless provides novel information about early Eocene fishes of North America and especially of the North Pacific Basin. It is a mixed fauna comprised of benthopelagic deep-water taxa (aff. *Chlamydoselachus*) and bathydemersal deep-water taxa (*Mitsukurina*, *Odontaspis*), open marine, epipelagic sharks (e.g., *Alopias*, *Brachycarcharias*, *Jaekelotodus*, *Macrorhizodus*, *Otodus*, *Striatolamia*), and at least one demersal, shallow marine shark (*Heterodontus*). Together, this assemblage corroborates paleoenvironmental interpretations for deposition of the lower Crescent Formation that were previously based only on microfossils and geology.
